# A Foodborne Outbreak of Gastroenteritis Caused by *Vibrio parahaemolyticus* and Norovirus through Non-Seafood Vehicle

**DOI:** 10.1371/journal.pone.0137848

**Published:** 2015-09-16

**Authors:** Yanhui Liu, Yat Hung Tam, Jun Yuan, Fengling Chen, Wenfeng Cai, Jianping Liu, Xiaowei Ma, Chaojun Xie, Chuangliang Zheng, Li Zhuo, Xianbang Cao, Hailing Tan, Baisheng Li, Huaping Xie, Yufei Liu, Dennis Ip

**Affiliations:** 1 Guangzhou Center for Disease Control and Prevention, Guangzhou, Guangdong, China; 2 Sun Yat-sen University, Guangzhou, Guangdong, China; 3 School of Public Health, University of Hong Kong, Hong Kong, China; 4 Foshan Chancheng District Center for Disease Control and Prevention, Foshan, Guangdong, China; 5 Guangzhou Luogang District Center for Disease Control and Prevention, Guangzhou, Guangdong, China; 6 The Eighth People’s Hospital of Guangzhou, Guangzhou, Guangdong, China; 7 Guangdong Provincial Center for Disease Control and Prevention, Guangzhou, Guangdong, China; Beijing Institute of Microbiology and Epidemiology, CHINA

## Abstract

Foodborne outbreaks caused by a mixed infection of *Vibrio parahaemolyticus* and norovirus have rarely been described. We reported a mixed outbreak of *Vibrio parahaemolyticus* and norovirus causing acute gastroenteritis in 99 staff members of a company in Guangdong, China, in May 2013, following consumption of roasted duck, an uncommon non-seafood vehicle for such mixed infection, in one meal served in the company's catering service. Epidemiological and laboratory findings indicated that a single asymptomatic food handler was the source of both pathogens, and the high rate of infection of both pathogens was exacerbated by the setting’s suboptimal food hygiene practice.

## Introduction

*Vibrio parahaemolyticus* (VP) and norovirus (NV) can cause foodborne outbreaks in various settings that usually emerge as self-limiting acute gastroenteritis which resolves within a couple of days [1–4]. VP-associated gastroenteritis typically occurs 4–96 hours after consuming contaminated food with symptoms that include diarrhoea, abdominal pain, nausea, vomiting, headache, fever and chills, lasting up to three days [1]. VP outbreaks associated with consumption of raw or improperly cooked seafood or salted food commonly occur in summer [5–6]. VP generally appears in freshly harvested seafood at a level below the predicted dose to cause infection, and is extremely sensitive to heat [6,7,8]. However, at ambient temperature, the organism can multiply rapidly to a sufficient infectious dose [1]. On the other hand, NV infection may occur throughout the year and can cause outbreaks in household and institutional settings, with restaurants being the most common setting of foodborne outbreaks [3–4]. Infectious food handlers have been implicated as the source of contamination in 70% of the NV outbreaks in the United States with their source of contamination reported [3]. NV infection usually incubates for 10 to 51 hours before causing vomiting, abdominal cramps, fever, watery diarrhoea, headache, chills and myalgia, which normally last for 2–3 days [4]. NV is very contagious due to its very low infectious dose, while subclinical infections can occur in some individuals [9–10]. A foodborne outbreak caused by VP and NV simultaneously was reported in Spain once after causing diarrhoea in three people who consumed bivalve molluscs [11]. Here we report the field investigation results of a large-scale gastroenteritis outbreak of mixed VP and NV infection in China carried by a vehicle other than seafood.

## Investigation Methods

The Guangzhou Center for Disease Control and Prevention was notified of a suspected foodborne outbreak on May 21, 2013, involving more than 50 staff members of a local company who went to a hospital in Guangzhou city with gastroenteritis on the evening of May 20. Active case finding was done through face-to-face or phone interviews using a company staff list and matching with medical consultation records of all hospitals located within 2 km of the company or its staff dormitories. “Suspected case” was defined as any staff member of the company who developed diarrhoea (≥3 times per day) or vomiting since May 18, 2013. A confirmed case included anyone whose stool specimens or anal swab samples were tested positive for VP or NV by polymerase chain reaction (PCR). As different meals were regularly served to all staff in the canteens on different floors of the company, a retrospective cohort study was conducted to identify the potential culprit meal(s) by comparing the attack rates (AR) among all staff members for each meal on different floors of the canteens during a period of three days (May 18–20) and relative risks (RR) among those who consumed the meal against those who did not. History of consumption of different meals was confirmed by the attendance recording system in the canteens for serving free meals to staff. A questionnaire survey was then carried out for all cases and controls selected from all staff to identify the food item(s) potentially contributing to the outbreak. The same number of controls were randomly selected from all staff members who attended the identified culprit meal but remained asymptomatic. By using a case-control study design, odds ratios (OR) of consuming different food items for all and laboratory confirmed cases versus selected controls were calculated. We collected stool, vomitus, and anal swab samples of the cases; anal swabs of cooks and waiters; environmental swab samples of the chopping boards for raw and cooked food, bench surfaces, cooking utensils, refrigerator, sink basin for washing vegetables and food trays in kitchen; and residual food samples for laboratory diagnosis. We analysed all demographic and epidemiological data using SPSS 13.0. A p-value <0.05 was regarded as statistically significant. This investigation, including collection of patient information and specimens, was undertaken in response to a public health emergency. Requirement of patient consent was waived by the Ethics Committee of the Guangzhou Center for Disease Control Prevention.

## Laboratory Methods

We tested all specimens for common pathogenic enteric bacteria (including VP, *Vibrio cholerae*, pathogenic *Escherichia coli*, *Staphylococcus aureus*, haemolytic streptococci, *Proteus spp*., *Salmonella spp*., *Shigella spp*. and *Bacillus cereus*) using bacterial culture and identifying VP by PCR. For bacterial culture, specimen was inoculated into 3% NaCl alkaline peptone water and incubated at 37°C for 16 hours, then was streaked onto thiosulphate-citrate-bile salts-sucrose agar plate for further incubation at 37°C for 24 hours for isolation of VP. Three suspected colonies were then selected and inoculated onto 3% NaCl tryptone soy agar and incubated at 37°C for a further 24 hours for pure culture, which was then tested for oxidase reaction, halophilia and biochemical identification. To prepare bacterial DNA for PCR identification, pure culture of suspected VP was inoculated into 3% chlorinated brain heart infusion broth and incubated at 37°C overnight, then centrifuged at 10000 rpm for 10 minutes. The precipitate was washed twice with sterile normal saline and resuspended in double-distilled water for boiling for 10 minutes. The bacterial lysate containing bacterial DNA was then put through PCR. Two μL of lysate, 1 μL of 20 μmol/L primer containing solution, 1 μL of 2.5 mmol/L dNTP containing solution, 0.3 μL Taq DNA polymerase (TaKaRa Company) and 5 μL of 10X reaction buffer were put into reaction tube and out through PCR with initial denaturation at 94°C for 5 minutes, followed by 94°C for 1 minute, 55°C for 1 minute and 72°C for 1 minute for 30 cycles, and final extension at 72°C for 5 minutes. The sequences of primer were *tlhF* 5'-AAAGCGGATTATGCAGAAGCACTG-3' and *tlhR* 5'-GCTACTTTCTAGCATTTTCTCTGC-3' targeting the 450 base-pair fragment of *tlh* gene of VP. All reactions were run with positive and negative DNA controls. PCR products were then run in 2% agarose gel electrophoresis in 0.5X TBE buffer solution with constant voltage of 180V for 20 minutes and recorded by photography.

VP isolates were then serotyped according to K- and O-antigens. Bacterial suspension was obtained by cultivating pure inoculum on 3% NaCl tryptone soy agar at 37°C for 24 hours and then the culture was washed out with 3% NaCl 5% glycerine solution. A part of the suspension was tested against antisera for K-antigens (Tianjin Biochip Corporation) for agglutinating reaction. Another part of the suspension was subjected to high pressure at 121°C for one hour. The suspension was then centrifuged at 4000 rpm for 15 minutes; the supernatant was discarded and washed with normal saline, and the process was repeated three times before the suspension was tested against antisera for O-antigens (Tianjin Biochip Corporation) for agglutinating reaction. The O-antigen type was defined as unknown if the suspension remained agglutination-negative with the O-antisera after retreating with high pressure at 121°C for one hour again. Multilocus sequence typing (MLST) on a sample of VP isolates was also performed for 7 housekeeping genes (*recA*, *gyrB*, *dnaE*, *dtdS*, *pntA*, *pynC* and *tnaA*) of VP. The VP isolates were also typed by using pulsed field gel electrophoresis (FMC's SeaKem Gold PFGE agarose gel, CHEF Mapper, Eppendorf's BioPhotometer) with endonucleases NotI (NEB) and protease K (Merck) run according to PulseNet operation protocol for VP. The gel was then stained by using ethidium bromide solution and photographed (Biorad Gel Doc 2000) and analysed (BioNumerics version 6.0).

Random samples of stool specimens from 20% of cases were also tested for viruses commonly causing gastroenteritis (i.e. NV genogroups I & II, astrovirus, rotavirus, and enteric adenovirus) using ELISA (R-Biosphere) and for NV using real-time fluorescent reverse transcription PCR (RT-PCR, Qiagen Viral RNA Mini Kit for viral RNA extraction). The sequence of primer for RT-PCR was targeting a conservative region of ORF1 gene common to NV genogroups I & II (DaAn Gene Co. Ltd.).

## Investigation Results

**Fig 1:**
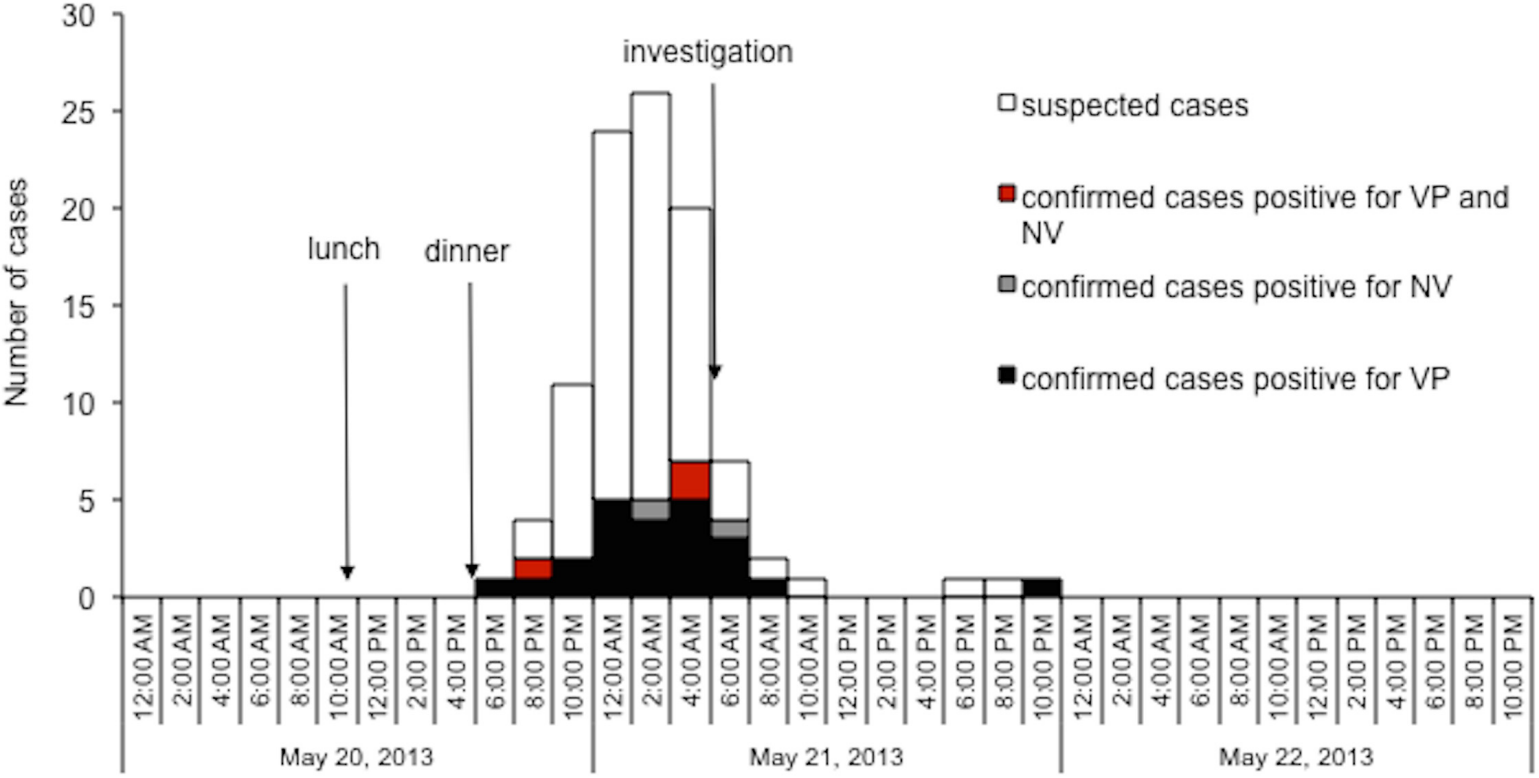
Epidemic curve of the outbreak showing the 99 cases of staff members

The involved company had 2049 employees (1303 male and 746 female). A total of 99 staff members were identified as fulfilling the suspected case definition, resulting in an overall attack rate of 4.83% for all staff. Their ages ranged from 18 to 50 years with a male to female ratio of 1.06:1. All cases had symptom onset between May 20–21, with most (69.70%) occurring between 0:00 AM and 6:00 AM on May 21, giving a unimodal pattern for the epidemic curve (Fig 1). Presenting symptoms included diarrhoea (100%, 85.9% were watery), abdominal pain (93.9%), fatigue (61.6%) and vomiting (54.55%), dizziness (45.5%), fever (20.2%), headache (14.1%), with symptoms lasting between 1–6 days (27.3% of them recovered within 2 days and 90.9% recovered within 3 days). Among those 50 staff members who had blood tested for white cell count, 84.0% showed leucocytosis with 80.9% being neutrophil predominate. Forty-one of them (41.4%) were hospitalised without serious complications or mortality.

Most employees lived in staff dormitories but no aggregation of cases by dormitory address was noticed. Lunch, dinner and midnight meals were regularly provided to all staff in the canteen on three different floors of the company building. Among meals served on different floors during the period of May 18–20, the lunch served on the second floor on May 20 was the only meal attended by all cases and had the highest AR (15.37%; 4.35% for confirmed cases) among attendees, giving rise to a RR of infinity (p <0.0001) when compared with staff who had not consumed that meal. No statistically significant positive RR was revealed for other meals served during the period (Table 1).

**Table 1:** Attack rates and relative risk of gastroenteritis among 2049 staff members having meals on different floors of the company canteen, May 18–20. Notes: 1. *Fisher exact test; 2. Figures in brackets referred to confirmed cases

			Exposed staff			Non-exposed staff				
Date	Meal	Floor	No. cases	No. total	Attack rate (%)	No. cases	No. total	Attack rate (%)	RR	95% CI or p-value
May 20	lunch	first floor	0	404	0	99 (26)	1645	6.02	0 (0)	P<0.0001*
	lunch	second floor	99 (26)	644	15.37	0	1405	0	+∞ (+∞)	P<0.0001*
	dinner	first floor	16 (3)	550	2.91	83 (25)	1499	5.54	0.53 (0.33)	0.31~0.89
	dinner	second floor	35 (12)	582	6.01	64 (16)	1467	4.36	1.38 (1.89)	0.92~2.06
May 19	lunch	first floor	5 (1)	373	1.34	94 (27)	1676	5.61	0.24 (0.17)	0.10~0.58
	lunch	second floor	23 (7)	599	3.84	76 (21)	1450	5.24	0.73 (0.81)	0.46~1.16
	dinner	first floor	11 (3)	475	2.32	88 (25)	1574	5.59	0.41 (0.40)	0.22~0.77
	dinner	second floor	11 (2)	374	2.94	88 (26)	1675	5.25	0.56 (0.34)	0.30~1.04
May 18	lunch	first floor	12 (2)	419	2.86	87 (26)	1630	5.34	0.54 (0.30)	0.30~0.97
	lunch	second floor	22 (7)	658	3.34	77 (21)	1391	5.54	0.60 (0.70)	0.38~0.96
	dinner	first floor	14 (4)	512	2.73	85 (24)	1537	5.53	0.49 (0.50)	0.28~0.86
	dinner	second floor	11 (4)	419	2.63	88 (24)	1630	5.40	0.49 (0.65)	0.26~0.90

While 99 controls were randomly selected among those remaining 545 asymptomatic staff members who had also attended the lunch served on second floor on May 20, only 55 of them could be successfully contacted for case-control study because the company refused to offer further assistance. Comparing all 99 cases with those 55 controls about consumption of food items (roasted duck, fried yardlong bean, braised tofu, lettuce, radish soup, sour bean and fried pickled radish) in that lunch on May 20, roasted duck was found to be associated with both suspected cases (OR = 4.94) and laboratory confirmed cases with VP (OR = 7.41) (Table 2). Eleven other cases that did not have roasted duck had the sauce served in the dish of roasted ducks. A dose-response relationship between the amount of roasted duck consumed (1 to >3 quarters of a duck) and the risk of gastroenteritis was demonstrated (χ^2^ for trend = 4.71, p = 0.03).

**Table 2:** Odds ratios of consuming different food items among cases with gastroenteritis and confirmed *Vibrio parahaemolyticus* (VP) infection versus randomly selected controls for the lunch on May 20, 2013. Remarks: 1. some subjects forgot the food items consumed. 2. Data in square brackets were of the cases positive for VP only.

	All cases (n = 99) [Confirmed VP cases (n = 26)]		Controls (n = 55)			
Food item	exposed	unexposed	exposed	unexposed	OR	95% CI
roasted duck	88[24]	11[2]	34	21	4.94[7.41]	2.01~12.35[1.46~50.54]
≥3/4 roasted duck	54[12]	0	15	0	χ^2^ for trend = 4.71[1.4]	p-value = 0.03[0.24]
1/2 roasted duck	21[9]	0	8	0		
1/4 roasted duck	13[3]	0	11	0		
fried long bean	85[22]	13[3]	43	12	1.82[2.05]	0.71~4.71[0.46~10.26]
braised tofu	78[20]	19[5]	39	15	1.58[1.54]	0.68~3.69[0.44~5.69]
Lettuces	82[25]	16[1]	43	12	1.43[6.98]	0.57~3.55[0.85~152.09]
green radish soup	57[19]	31[6]	38	11	0.53[0.92]	0.22~1.27[0.26~3.32]
sour bean	39[10]	54[15]	15	38	1.83[1.69]	0.84~4.04[0.55~5.15]
salted green radish	11[6]	78[20]	3	50	2.35[5.00]	0.57~11.22[0.97~28.47]

Twelve food handlers involved in preparing the incriminated meal denied any recent symptoms of gastroenteritis. The roasted ducks served at that meal were bought from a local shop on the morning of May 20 and further chopped and sauced in the kitchen before being served during the lunch time more than four hours later, without being reheated before serving as was the case for other food items. As of May 25, no similar cases were reported from customers of the other 20 restaurants that had bought roasted ducks from that local shop on May 20.

Twenty-six cases were tested positive for VP and 5 cases for NV from one or more of their clinical specimens (stool, anal swab or vomitus); 3 of them were positive for both. The 5 cases positive for NV were residing in different rooms and floors of the dormitories and working in different offices. Aside from the common meal on May 20, the 5 cases positive for NV did not share toilets and had no contact with each other within the three days of their symptom onset. No other relevant food history including consumption of seafood or unboiled water could be elicited from these 5 cases. Anal swab samples from 2 food handlers were positive for VP, including one (food handler A) who was also positive for NV and had handled the roasted ducks with his bare hands. He handled raw freshwater fish in the same kitchen a few days earlier, but he denied consumption of the roasted duck or any seafood product recently or contact with anyone known to have gastrointestinal illness. Another food handler positive for VP was responsible for preparing the roasted duck sauce and ate the roasted duck on May 20. While the symptom profile of VP and NV cases considerably overlapped with each other, significantly more severe vomiting was observed among NV cases (3–8 times, median 6) than VP cases (0–3 times, median 1) (Mann-Whitney U test, p = 0.0002), and longer duration of symptoms among NV cases (1–6 days, median 4 days) than VP cases (1–3 days, median 3 days), though not statistically significant (Mann-Whitney U test, p = 0.07).

**Fig 2:**
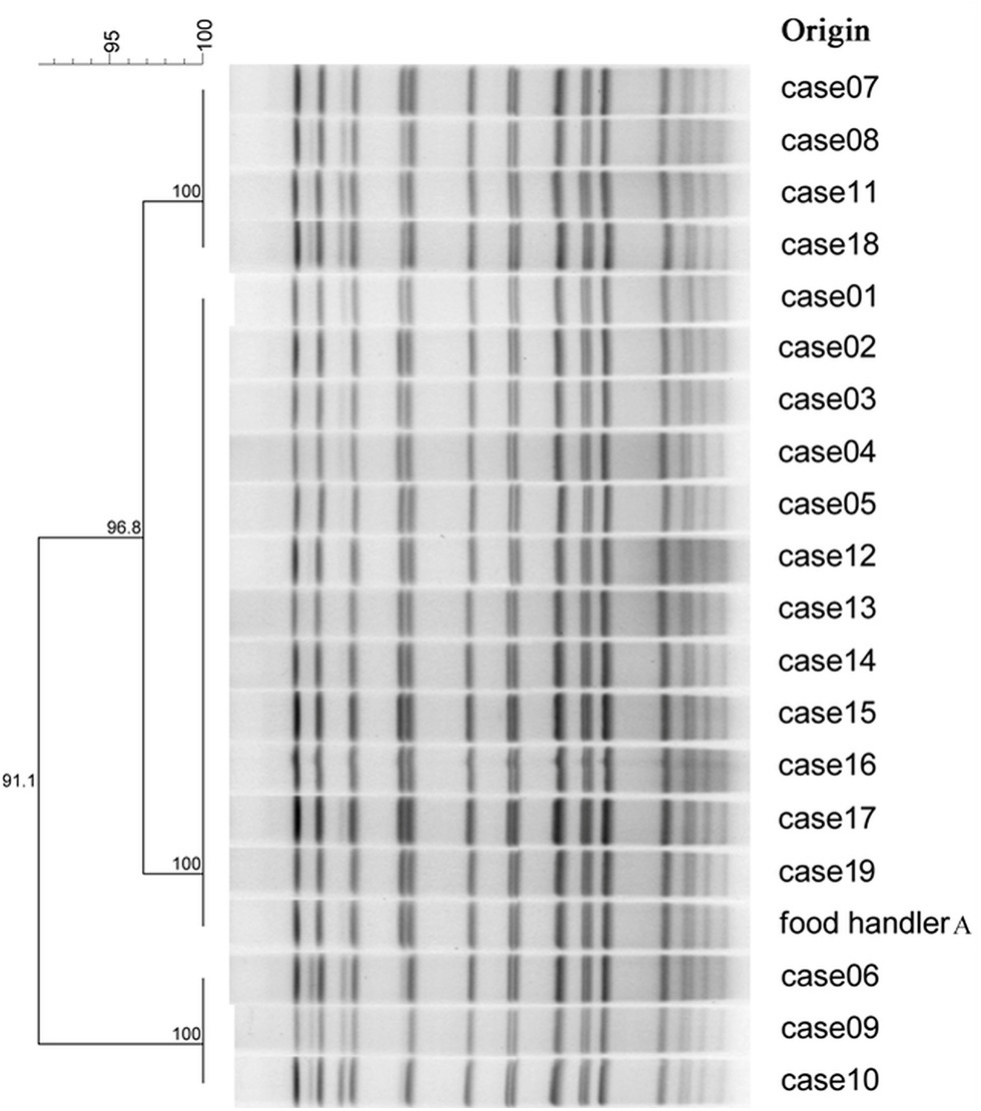
Pattern of pulsed-field gel electrophoresis of *Vibrio parahaemolyticus* (VP) from 19 cases and food handler A with culture-positive specimens. The pattern from the specimen of food handler A showed 91.1–100% homology to those from 19 cases.

VP was successfully cultured from the PCR-positive samples of cases and food handlers and all belonged to serotype O3:K6. Specimens from 3 cases and food handler A were further typed to be ST3 by MLST. PFGE for the culture-positive specimens of 19 cases and food handler A revealed their 91.1–100% homology (Fig 2). Out of 13 samples of residual food, only a roasted duck sample was tested positive for VP. None of the 13 environmental samples tested positive.

## Discussion and Conclusion

This foodborne outbreak of gastroenteritis was likely contributed by both VP and NV through contaminated roasted duck. VP is recognized as one of the major causes of foodborne diseases in Guangdong [12] and 0.3% of healthy people were reported to be VP carriers [13]. Food handler A could have been an asymptomatic carrier and contaminated the roasted ducks. The 4-hour timeframe that the prepared roasted ducks sat before being served might have allowed VP to multiply. Other foods could have been similarly contaminated, but their reheating before serving might have prevented them from transmitting the pathogens. The common source of VP for the case and food handler A was highly suggested by the similarity of their VP isolates in terms of serotype, sequence type and PFGE results. Although we could not culture the VP identified from the roasted duck sample for MLST and PFGE to confirm its homology with those VP isolated from food handler A and the cases, the combination of epidemiological and laboratory findings suggested that the roasted duck was the most likely VP-transmitting vehicle leading to this outbreak.

The positive rate of NV was 23.81% (5/21) among the cases. Although this rate may be overestimated due to testing on only some selected cases, it was still much higher than the carrier rates of NV reported among adults (2.81%) in Vietnam and food handlers (3.42%) in China [9–10] if taking random selection among all cases into consideration, supporting the causative role of both VP and NV in this outbreak. Moreover, the discernible differences of symptomatology between VP and NV cases suggested that NV infection was more likely to be part of the outbreak rather than an incidentally found by-stander. However, given that 41% of cases required hospitalization, most of the cases were likely affected by VP with NV playing a relatively minor role to the severity in this outbreak. The lack of geographical clustering among the five NV cases in their dormitory further supported their foodborne acquisition instead of person-to-person transmission of NV. Food handler with subclinical or asymptomatic infection can transmit NV through processing deli [14] or bread products [15], which is what the food handler A possibly did. Moreover, as individuals with asymptomatic NV infection had similar viral load to those symptomatic ones [16], asymptomatic food handlers may infect more people before they are removed from work than symptomatic ones and may be more likely to cause large outbreaks. However, our case-control study could not show roasted duck as the culprit food for NV infection based on the small number of cases confirmed for NV. Testing the roasted duck sample for NV, if it were possible, might help to suggest the chain of transmission.

This outbreak demonstrated that the amounts of VP and NV shed from a reportedly asymptomatic food handler were sufficient to cause a large outbreak through contamination of ready-to-eat food that was not properly reheated. As VP and NV may induce only mild symptoms in some individuals, infectious food handlers may not recognize their own infection and thus cannot be relied on to remove themselves from work on time. Monitoring and regulation of any violation of proper food handling processes in the food catering industry should be the mainstay of preventive measures to be strengthened.
